# Exploring pandemic metaphors in educational contexts: a survey on the language of teachers and educators in Reggio Emilia, Italy

**DOI:** 10.3389/fpsyg.2023.1192653

**Published:** 2023-05-16

**Authors:** Alice Giuliani

**Affiliations:** Department of Education and Humanities, University of Modena and Reggio Emilia, Reggio Emilia, Italy

**Keywords:** war metaphors, language in education, pandemic (COVID-19), metaphor theory, creative metaphors, pragmatic implications

## Abstract

This study aimed to explore how metaphors were used to interpret the pandemic and to address its challenges in primary and secondary schools in Reggio Emilia, Italy. A questionnaire was administered to educators and teachers to understand how languages, images, and metaphors were used by themselves and their students to talk about the pandemic and their experiences of living with it. The goal of the questionnaire was to guide critical reflection and encourage more informed language choices. While the existing literature points out the alleged overuse of war metaphors and military frames in public discourse, our findings show that war metaphors are relatively frequent, with other metaphorical frames widely used by teachers and educators to foster resilient attitudes in students. Moreover, in their professional contexts, teachers and educators mostly use metaphorical frames involving resilient attitudes. Our interpretation of the results supports the hypothesis that the purposeful use and deliberate production of metaphors support the choice of metaphors with positive, constructive implications. Finally, some implications of these findings on the theory of metaphor and the methodology of the research are discussed.

## Introduction

Metaphors have been considered powerful devices in such areas as medicine and healthcare communication, with scholars investigating their role as cognitive tools to acquire knowledge, share illness experiences, and promote healthy behaviors (Gibbs and Franks, 2002; Nie et al., 2016; Semino, 2018; Macagno and Rossi, 2019; Hauser and Schwarz, 2020). This topic has also found resonance in the public and cultural debate, especially regarding the issue of the “war on cancer” (Sample, 2019). The best-known reference in the debate on the use of metaphors to discuss illnesses is the essays by Susan Sontag (1978, 1989), who had led more generally (as noted in Piazza, 2022) a critical discussion of the consequences of language choices on the experience of the sick person.

Beginning in 2019, with the spread of the Coronavirus pandemic, the use of the metaphor of war has been the focus of widespread discussion for many months. Numerous interventions, critical of the “war on the virus” metaphor, have been published in newspapers and journals (i.e., Craig, 2020; Tisdall, 2020, in Italy, Battistelli, 2020; Costa, 2020; Lingiardi and Giovanardi, 2020; Semino, 2020; Solidoro, 2020; Sturloni, 2020; Testa, 2020).

The primary focus of studies on the potential dangers of using war metaphors to discuss the pandemic has been on public communication. The need to apply emergency measures with a serious impact on the community has increased the importance of public communication, and scholars have noted extensive use of military metaphors by institutions and politicians.

«Esta guerra porque de uma verdadeira guerra se trata dura há um mês, começou depois dos vizinhos europeus, e, também por isso, pôde demorar mais tempo a atingir os picos da sua expressão» (Marcelo Rebelo de Sousa, 18 March 2020).

« Nous sommes en guerre […] J’appelle tous les Français à s’inscrire dans cette union nationale […]. Nous sommes en guerre, oui. […] Hissons nous, individuellement et collectivement, à la hauteur du moment» (Emmanuel Macron, 12 March 2020).

«Every generation of Americans has been called to make shared sacrifices for the good of the nation. To this day, nobody has ever seen what they were able to do during World War II.

Now it’s our time. We must sacrifice together because we are all in this together, and we will. Come through together. It is an invisible enemy. That is always the toughest enemy, invisible.

enemy» (Donald Trump, 18 March 2020).

Scientific studies, preceding the pandemic, have already documented the widespread use of war metaphors in public discourse (Karlberg and Buell, 2005; Flusberg et al., 2018). In recent years, new studies have discussed the pervasiveness of the war metaphor for discussing the pandemic in public discourse (Benziman, 2020; Gillis, 2020; Castro Seixas, 2021). Additionally, Wicke and Bolognesi (2020) recorded its application in the language used in social networks, where the war framework was the most commonly utilized option among the figurative frames. Several studies have provided support for the critical stance toward the use of this metaphor, for several reasons. The metaphorical frame was judged to be inappropriate and reductive to the complexity of the pandemic. In other cases, it is regarded as the cause of cognitive misunderstandings, as it shifts the focus away from aspects that are important for understanding the reasons for the phenomenon and its prevention (Farruggia, 2020). Other scholars believe that it is not effective in reinforcing positive (empathetic, supportive) reactions and attitudes, and that other metaphors “that make social cohesion and solidarity salient” should be preferred (Schnepf and Christmann, 2022). Some authors go to unconditional criticism: Bates (2020), for example, accuses the war metaphor of provoking a “rhetorical incoherence and undermine policy response to SARS-CoV-2” and calls for the rejection of war as a metaphor for understanding COVID-19 (for a review of motivations in the literature, see Olza et al., 2021; Panzeri et al., 2021).

The arguments for rejecting the metaphor of war seem to share deterministic assumptions. Such a presupposition may be derived from a radical cognitivist interpretation of Lakoff and Johnson’s studies, according to which metaphoric mapping is grounded in predetermined conceptual structures and has the effects of rigid and unambiguous selection in the receiver’s beliefs. Bates (2020), for example, understands Lakoff and Johnson (1980) and Lakoff (2004) to argue that metaphor acts as a “strong frame” because “the very structure of cognition *may cause* speaker and auditor to view the entailments of the metaphors as being true” (my emphasis).

Contrarily, the idea of deterministic framing of metaphors, and war metaphor in particular, has been discussed and reviewed in both theoretical and empirical studies. An important reference is Flusberg et al. (2018), who partially corrected some previous studies and proposed a contextual perspective, according to which the meaning and consequences of war metaphors are intimately tied to the context in which they are used. Thus, the authors warn against the blanket statements about the war frame since they are misguided or overly constraining: “the fear evoked by war metaphors can be de-motivating, but the war metaphor may also be useful in encouraging preventative behaviors” (Flusberg et al., 2018, p. 7; *cf.* Piazza, 2022). Recent studies in sociology, philosophy, psychology, and semiotics have followed the same lines. For example Marino (2021), calls for acknowledging that war metaphors often do not evoke concepts or images of the literal domain of war and do not stimulate real projections or interactions between concepts (*cf.* Bolognesi, 2021). The author calls for practicing “interpretive charity” toward this linguistic use, questioning the prevalence of the war metaphor without risking presupposing its reasons and consequences. In the field of empirical studies, Panzeri et al. (2021) argued experimentally that the willingness to accept war-congruent claims about the pandemic is not directly influenced by war metaphoric framing *per se*, and is favored by socio-political individual variables and sources of information (Panzeri et al., 2021, p. 11). Analyzing various empirical studies, Benzi and Novarese (2022) concluded that the current evidence does not support the claim that the use of metaphor can lead citizens to accept limited civil liberties and authoritarian policies. Among the studies reviewed, they mention the study on Twitter communication by Wicke and Bolognesi, which attests that war framing is indeed often used to talk about specific topics, such as the treatment of the virus, but not others, such as the effects of social estrangement on the population. The use of the frame thus seems consistent with limitations that, according to critical scholars, are always on the point of being ignored and exceeded in linguistic practice. Benzi and Novarese consequently agree with Flusberg et al. (2018) about the importance to not assume “a preconceived attitude on the use of the metaphor,” thus assigning misleading power to the frame: they consider the unconditional criticism of the use of the war metaphor unfounded and choose other options than rejection of the metaphor itself.

However, some of these studies, critical of the deterministic action of frames, remain aligned with some of the premises of the Conceptual Metaphor Theory. For instance, Panzeri et al. (2021) accept that war functions as a “structural metaphor” in Lakoff and Johnson’s (1980) terms, since there are several correspondences between the cluster of notions of the source domain “war” and the notions that might be associated with the target domain “pandemic”: e.g., “the virus and an enemy; health professionals and an army…eliminate the virus and victory” (Semino, 2020).

The current study aimed to explore the metaphorical language used to talk about the experience of living with the pandemic in primary and secondary schools in Reggio Emilia (Italy). The framework of this study partially adjusts the CMT perspective with the concept of interaction, which was firstly defined and developed by Black (1955, 1977) and more recently resumed and enhanced in studies on multimodal metaphors by Forceville (1998, 2016), transdisciplinary studies on metaphor in communication (i.e., Gola and Ervas, 2016) and philosophical studies on metaphors and creativity (Contini, 2018). According to Black’s perspective, metaphorical expression does not merely reflect and express linguistically a structural correspondence between the concepts involved in the projection, but rather helps to establish, to enable correspondence. As Ricoeur wrote (1975), “resemblance,” in the sense of appearing similar, is (also) a product of metaphor: a human being, for example, does not have the same features as a wolf, except for metaphorical transposition, and resemblance is an act, a process enacted with the resources of the imagination. According to the interaction view, there is no reason to argue *a priori* that the use of war words (“soldiers,” “alert,” “front of war”) determines the framing of the pandemic as “war,” or is indicative of the implementation of a military conceptual structure. Instead, it can be argued that the use of a metaphor is effective based on analogy and underlying conceptual correspondences, but that the implications and meanings produced depend on the context of use. Thus, metaphors of war (i.e., “the need for everyone to mobilize and do their part on the home front”), can work to effectively communicate the need for “taking social distancing orders and hand washing recommendations seriously” (Levenson, 2020) without this usage disposing a structured and unambiguous framing of the pandemic domain.

Based on this perspective, it becomes important to value the role of the receiver as the interlocutor in a communication (Piazza, 2020; Steen, 2008) and to devote adequate attention to the “emphasis” function of metaphor (*cf.* again Black, 1977, pp. 440–441) and its capacity, of Aristotelian memory, to present, to “put before the eyes” and “make seen” (Ricoeur, 1975, Aristotle, Rhetoric 1410b: 32–34). The interactive view calls us to remember that the cognitive function of effective metaphors, capable of making a concept thinkable and significant, depends on an “insight” that is a function of imagination. “Good” metaphors are endowed with emphasis as well as resonance (they support a high degree of implicative elaboration) (Black, 1977, pp. 439–440). Among them, strong metaphors are also “necessary” metaphors: they respond to the need to embody an insight that is not otherwise expressible (Black, 1977, p. 448, Giuliani, 2023). It follows that in order not to reduce metaphor to the expression of an underlying conceptual structure, it is important to shift the focus from understanding to producing metaphors: to ask what it means, for the producer, to see one thing as another and (to have to) think of something as something else (Black, 1977, p. 446).

Empirical research on metaphors used to discuss the pandemic thus seems more reliable and relevant when considering the variables of context and relationship between interlocutors (i.e., Chen et al., 2021, with the focus on people with disabilities; for the contextual theory, Kövecses, 2005; Gibbs, 2017). However, few studies have examined the use of metaphors for pandemics in educational contexts. Świątkiewicz-Mośny et al. (2022) conducted a study in the field of health literacy (HL) and analyzed 247 educational materials from different countries dedicated to children (and also adolescents, and their carers) explaining the pandemic. Attention was also paid to the nomenclature and metaphors applied to describe the virus and the situation resulting from its spread (“war,” “struggle,” “monster,” “players,” “heroes”). Similar to our study, other scholars have analyzed the metaphors used and produced by teachers to better understand their experience of the pandemic, especially focusing on distance learning. Among Italian studies, the research by Troina et al. (2021) similarly dealt with the ways teachers experienced the condition of distance teaching, and metaphors were analyzed to better understand the feelings described by participants. The documented results show that “many participants felt that this change was non-reversible, as future scenarios will always have to come to terms with what happened during the pandemic period.” In Turkey, Sipahioglu (2022) identified and classified the metaphors produced by a sample group of teachers to understand their perceptions of distance education during the COVID-19 pandemic, and to suggest better policies to implement during emergencies. Working with a different and proactive approach, in the USA, Anderson et al. (2021) focused on “the role of metaphor in creative teaching and learning, especially in making sense of and managing the stress of crises and uncertainty.” Teachers were supported by metaphoric and narrative resources (“metaphors cards”) and other training and materials designed to innovate creative learning routines. The authors found that “creative self-efficacy in teaching is related to teacher buoyancy in the face of setbacks, such as distance learning.”

However, research on the use of metaphors in education finds ample space in cognitive studies in science education. Recent studies argue that teachers’ conscious use of metaphors and students’ analysis of conceptual metaphors are important in science learning (Lancor, 2012, 2015; Amin, 2015). This exhortation is based on a conception of abstract concepts as an integration among many elements, also including iconic representations by imaginative simulation (Amin, 2015, p. 6). Referring also to Carey (2009) on conceptual development, Amin argues that scientific understanding is not the sequential correction of errors and misconceptions, but rather a process of conceptual change through which conceptual networks, prior to exposure to instruction, are transformed into conceptual structures consistent with the knowledge of the “expert” scientist (Amin, 2015, p. 8).

In learning thought as conceptual change, metaphors become important for two main reasons: on the one hand, metaphor is the propositional tool that activates the schema-images necessary for understanding concepts; on the other hand, conceptual metaphors based on sensorimotor schema-images are effective devices for integration that enable the formation of concepts (Amin, 2015, pp. 8–9). Consequently, identifying the metaphors used by students is useful for analyzing and breaking down the concepts they use and recognizing misconceptions, while the critical use of metaphors by teachers is important for consciously guiding the processes of conceptual change, including the use of imagination. Lancor, for his part, highlights that every scientific concept undergoes metaphorical mapping, and conceptual metaphors understood by our society at large have a significance that depends on the particular context in which they are employed. For example, many metaphors for energy represent different conceptualizations of energy created in a given social context. However, his conception goes further: metaphors are not just heuristic tools for understanding a concept or framing devices for different aspects of a knowledge object. According to Lancor, following the notion of creative metaphor in the philosophy of science (Black, 1962; Hesse, 1966), there is often not a single, overarching concept (such as a concept of energy) in science that is explicated using multiple metaphors. Rather, the definition of the concept emerges as a result of negotiation that occurs in diverse contexts of situated cognition. The absence of a comprehensive, all-encompassing definition of the concept is thus not a limitation of our understanding, but a constitutive aspect of scientific concepts for which “a functional, context-dependent and metaphorical understanding is the best we can do.” Recent studies by scholars from the research centre “Metaphor and Narrative in Science” of University of Modena and Reggio Emilia, in line with previous statements from philosophical and pedagogical studies (Ervas et al., 2017; Egan, 1990, 2019), argue that the processes of understanding and producing metaphors, along with storytelling, allow for the exercise of “imaginative rationality” which is a fundamental resource of learning as relationship building (Fuchs et al., 2018; Contini, 2020; Giuliani and Manera, 2022).

## Materials and methods

### Design of the study

The use of language in educational contexts during the pandemic was the topic of the project “The language of the pandemic in educational contexts,” which between November 2020 and March 2021 involved about 200 teachers, educators, and pedagogists from Reggio Emilia. The project was conducted by a research group from the Department of Education and Human Sciences, together with Maria Grazia Rossi (Universidade Nova de Lisboa) and with the collaboration of Officina Educativa, an institution that coordinates municipal educational services for the 6–14 age group of Reggio Emilia. A questionnaire was the research tool used to explore the language of the pandemic in local educational settings. Queries were posed to collect data on the vocabulary employed by teachers, educators, and students in reference to their encounter with the pandemic. Specific attention was devoted to the deliberate and non-deliberate use of metaphors (implicit choices were also considered; for example, by asking what images were associated with the pandemic, some non-deliberate metaphorical uses were linked). The further purpose was to suggest critical reflections useful for enriching participants with new resources to deal with the pandemic in their professional context. Simultaneously, the questionnaire was designed to encourage critical reflection on language use.

The questionnaire includes 6 sections, each with its own objective:

- Describe the sample: Demographic information (age, gender, educational qualification, role, teaching subject/subjects, years of experience in the same role, school grade).
- Encourage reflection regarding their own experience of the pandemic: Collection of words and images that participants associate with the pandemic and that they have used and heard during daily activities with children/youth.
- Survey educational initiatives: Collection of educational initiatives designed specifically for pupils and aimed at discussing the pandemic experience.
- Analyzing the use of metaphors in the educational context and encouraging metaphorical associations: Proposing multiple-choice options to collect metaphorical expressions used during educational activities to discuss specific aspects of the pandemic (pandemic as general situation, COVID-19, the contagion, measures to limit the contagion, other people, doctors and health care workers, the vaccine, the end of the pandemic).
- Encourage critical reflection on language choices: Collection of reflections on the meaning of some words (e.g., distancing, care, relationship, space) that gained relevance and resonance during the pandemic.
- Encourage critical evaluation of metaphors and identification of preferred metaphors for future use, comparing previous answers: collection of comments on the metaphors proposed in the questionnaire (negative, interesting and/or creative, and educationally effective metaphors).

### Classification of metaphors

In Section D, participants were asked to select the three metaphors used most often and/or most prominent to discuss the following aspects of the pandemic: the pandemic as an overall situation; COVID-19; the spread of the contagion, measures to contain the contagion, others during the pandemic, physicians and healthcare personnel active in COVID-19 care; the vaccine as a product of scientific research, and the end of the pandemic.

Participants were able to choose a maximum of three options from a set of metaphorical expressions. In the test construction phase, the first metaphors to be proposed were chosen based on the following criteria:

– Relevant Presence in Press Communication, Online Articles, and Social Networks. In particular, very frequent or problematic metaphors are subject to journalistic or scientific discussion.
– Original metaphors: unconventional associations.

The first metaphors chosen as response options were present in institutional communication on the pandemic issue and had become the subject of critical discussion: the war against the virus and the need to win the battle. Therefore, some frames were selected as counterexamples from the database of the #Reframecovid project (Olza et al., 2021), others from formal and informal communication contexts, and clarified them by articulating their conceptual features and possible pragmatic implications. In contrast to the conflict frame, some examples were identified based on the obstacle, problem, or game frame, whereas conflict-frame implies win/lose options and evokes the need to prevail by force, and the problem or obstacle frame foregrounds knowledge and strategy. In the former case, I have to defend myself and counterattack against a deliberately hostile action; in the latter case, the difficulty depends on my own limitations, which can be overcome by knowledge.

Some countertrends were found in scientific communication about the pandemic, where the virus was not referred to as an enemy to be defeated, but rather as a “*symptom*” of a broader ecological problem and imbalance, analogous to what happens with the symptom of a disease. Consequently, the metaphor of the virus as a *messenger* was introduced, intended as an *alarm* that must shift our attention and change our attitude.

To give expression to an entirely different pragmatic attitude of skepticism and distrust, some conceptual frames of the polemic narrative regarding political measures, such as lockdown, were chosen: constraint in the nuances of prison and dictatorship (with the metaphor, often not used as such, of sanitary dictatorship). Alongside this framework, which evokes a human-like exercise of power, the idea of a destructive but impersonal action, linked to the force of nature, was proposed: the category therefore of natural disaster, as an irreversible and destructive event but one that can at least be contained, limited, or finally neutralized by appropriate means and resources.

Alternatively, some options were also proposed based on the frameworks of madness, darkness, and nightmares, which appear to be united by the need to express an experience of totalizing negation: the idea of something fundamental failing, breaking down, an absolute negation with no apparent solution, and no precise agent.

Following a recursive procedure relying on subsequent adaptations, a variety of answer options for each of the pandemic features were defined for Section D. Moreover, some synonyms, experience, or pragmatic implications to clarify the meaning of the metaphor were added next to each option, in parenthesis. For example, for “monster”: “unseen, looming, scary”; for “fire,” it was clarified that it could be understood as an event “to be contained, whose damage is to be reduced.”

Listed below are all the options proposed for each feature-question:

Pandemic as an overall situation:

- storm (with ship at the mercy of waves, boat in danger of sinking),
- war (to fight, in which to eliminate the enemy…),
- match (to be won, in which to compete…),
- game (with rules, finding solutions, strategies…),
- night, darkness, nightmare (which must end, to be brightened…),
- madness (chaos, imbalance),
- revolution, transformation (opportunity for change),
- dictatorship (compromised freedoms, abuse of power, control),
- fire (spreading, compromising, destroying).

COVID-19 as:

- enemy, conqueror (to defend against, to react against),
- mountain (obstacle to overcome),
- alarm/messenger (waking us up, warning of global problems),
- blow/hammer/shock (that shakes, that knocks down, destabilizes),
- monster, ghost (unseen, looming, scary),
- opponent (in a game that has its own goal, on which we must prevail),
- flame (which seeks fuel, burns) / rain (which accumulates, finds cracks, infiltrates),
- explosive device (to be defused, rendered harmless).

The spread of contagion:

- fire (to be contained, whose damage to be reduced),
- military attack, military campaign (broad, spread over several fronts, with organized troops),
- avalanche, tsunami-flood (overwhelming, unpredictable, uncontainable…),
- train derailing (event to be prevented, depending on mistakes),
- domino, chain reaction, word of mouth (to be broken, disrupted),
- earthquake (which shakes ground underfoot, takes away stability, creates insecurity),
- colonization (of parasites, aliens…),
- river breaking banks (event to be prevented, dependent on mistakes).

Measures to contain contagion:

- pause, suspension, parenthesis (from the ordinary to reflect, return to self),
- seclusion, asceticism (revealing hidden, previously invisible things),
- role-playing/group work (collaboration),
- counterattack, resistance (to oppose, not to be annihilated, defeated),
- abyss, tunnel (absence of light, no exit in sight),
- shelter (in which to be safe),
- collective experiment (we are not sure of the results, we go by trial and error),
- prison (helplessness, absence of freedom, physical constraint).

Others during the pandemic:

- lead actors (everyone has mission, important role),
- threat/hunters/spies (someone to be wary of),
- allies (in the conflict against the virus),
- masks/aliens (we do not see their faces),
- companions (of adventure, travel, in the same boat),
- missing people (whom we have lost track of, whom we cannot meet),
- support, source of energy (to move forward, face difficulties, and start again),
- pawns (to be placed, organized in a strategy),
- puppets, marionettes (at the mercy of others’ decisions).

Doctors and medical staff active in the care of COVID-19:

- guides (who explore, lead us to the way out…),
- angels (who guard, protect…),
- machines (tireless, working tirelessly),
- victims (they sacrifice themselves for the collective good),
- new protagonists (who were in the shadows, who came to the foreground over other characters),
- heroes, superheroes (with above-average talent, capable of measuring themselves against abnormal events),
- judges (they decide life/death),
- stars (who love notoriety, who seek prominence),
- agents/special agents (dictated by the new power of medicine and science),
- soldiers (in the war on the virus).

The vaccine as a product of scientific research:

- counterattack weapon (against the attack of the virus to overcome its “troops”),
- trainer (to instruct our body to react),
- gatekeeper – filter (preventing the virus from hitting us, making it wait),
- way out, “esc” key (from the virus’ range of action),
- strategy, trick (to boycott the virus, weaken it),
- turning point (in the path of change initiated by the pandemic),
- neutralizer, tamer (which makes the virus less dangerous and allows people to live with it).

The end of the pandemic:

- liberation (from an invasion, occupation),
- salvation/victory (finding escape),
- return of light, miracle,
- oasis/mirage (which could be an illusion),
- rebirth, renewal,
- restart (after overcoming an obstacle),
- regaining freedom.

In the identification process of metaphors, the generic definition of metaphor as “understanding and experiencing one kind of thing in terms of another” (Lakoff and Johnson, 1980, p. 5) was adopted. For a more technical definition, it was preferred the idea of interaction between systems of implications and cross-domain mapping between the two conceptual domains (*cf*. Steen et al., 2010, p. 47). For the identification of metaphors in Sections B and F, some principles of the identification method developed by the research group of the University of Amsterdam were adopted. In particular, the following metaphors were considered:

- words used metaphorically, whose meaning is the indirect meaning of the word arising “from the contrast between the contextual meaning of a lexical unit and its more basic meaning, the latter being absent from the actual context but observable in others” (Steen et al., 2010, pp. 768–771).
- words expressing a conceptual domain that functions as a source domain in a mapping provided as some form of comparison (*cf.* Steen et al., 2010,, pp. 768–771).

To organize the data, a procedure was followed in several stages using a mixed (qualitative and quantitative) research design. Both the selected and produced metaphors were organized on the basis of different “categories of experience”: “experience” was intended as present experience, but also including attitudes and future-oriented experience. In describing these categories, the subject attitude (as a patient, agent, spectator, etc.) and the way the pandemic was experienced as an actor itself (indifferent, hostile, collaborative…) were considered. In doing so, six categories were obtained, including several frameworks that share similar pragmatic implications. Categories were distinguished by the type of experience and pragmatic projection. They can be arranged on a quantitative scale, based on the degree of negativity and indeterminacy, ranging from the experience of annihilation and indeterminate terror (NEGG), to that of positive interaction that prompts determinate innovation (TO).

The first category has been assigned the label NEGG.^1^ The frames include the concepts of nightmares, darkness, earthquake-shattering, and madness. The experience they are associated with is the feeling of being annihilated and completely lost. The pragmatic implication is the absence of any positive attitude, hopelessness, waiting for the end of the world, or life, as known before; however, the awareness of being dragged into something totally indeterminate.

The second category was assigned the label AROUND. The frames included in this category are the concepts of force of nature, disaster, and natural or supernatural catastrophe: they are all devastating, expanding events that are completely unpredictable and uncontrollable. The experience of the subject involved is a sense of being surrounded and isolated, resisting something, and oscillating between hopelessness and the attempt at containment and reconnection.

The third category is labeled ON, and marks the experience of oppression, coercion, and terror. Pragmatic projection is the search for an escape for liberation. The frames that have been included in this category are the concepts of jailer, chain, monster, and tyrant: some quite unknown agent, not completely determined from which we are willing to escape, or free ourselves.

The fourth category, labeled VS, includes frames of war, military strategy, and weapons. The subject’s experience is feeling attacked and forced to confront an enemy, an opponent with opposing violent purposes. The resulting principal attitude is an attempt to fight, prevail, and defend oneself.

The fifth category was identified using the FRONT label. In this case, the frames are the obstacle, mountain, problem, game, and match. The experience implied by these frames is the urgence to understand and solve a problem, to take part in some role-play, to collaborate, to solve some puzzling-strategic game, and to face an obstacle that is to be overcome. The attitude of the subject is a commitment to understanding, solving, and taking on a challenge.

The last category, the sixth category, was identified by the label TURN. The frames included in this category are concepts of signals, messages, and alarms. The pandemic is seen and experienced as a part of human history, as a consequence of human actions, preparing humanity for something new. The experience they make sense of is that of taking part in a story, taking a different direction on a path/journey, and getting involved in a mission. The suggested attitude is the action of listening, embracing, responding, and changing one’s attitude or perspective.

In the following table (Table 1) I have organized the response options into the aforementioned categories.

**Table 1:** Classification of metaphors in multiple-choice questions (Section D of the questionnaire).

	NEGG	AROUND	ON	VS	FRONT	TURN
The pandemic as overall situation	Night, darkness, nightmare madness	Storm fire	Dictatorship	War	Game match	Revolution transformation
The COVID-19	Blow/hammer/shock	Flame rain	Monster, ghost	Enemy, conqueror opponent explosive device	Mountain	Alert/messenger
The spread of contagion	Earthquake	Earthquake fire avalanche, tsunami-flood	Colonization	Military attack, military campaign	Domino chain reaction word of mouth	Train derailing river breaking banks
The measures to contain contagion	Abyss, tunnel	Shelter	Prison	Counterattack, resistance	Role-playing, group work collective experiment	Pause-suspension, parenthesis asceticism, seclusion
The others during the pandemic	Masks/aliens	Missing people	Puppets, marionettes	Threat/hunters/spies allies	Pawns support, source of energy	Lead actors companions
Physicians and health personnel	Angels sacrificial victims	Heroes, superheroes	Special agents judges star	Soldiers	Machines guides	(new) Protagonists
The vaccine	Way out, “esc” key	Neutralizer, tamer	[NONE]	Counterattack weapon	Trainer gatekeeper, filter strategy, trick	Turning point
The end	Return of light, miracle oasis/mirage	Salvation, victory	Liberation regaining freedom	Liberation salvation, victory	Restart salvation, victory	Rebirth, renewal

## Results

The questionnaire, digitized and offered anonymously, took participants up to 30 min to complete. A total of 122 answered. The majority of participants (55%) belonged to the 18–39 age group, 41% were between 40 and 59 years old, and the remainder were over 60 years old. Only 10 participants were men; it results a large proportion of women, which reflects the ordinary composition of personnel in educational services. Approximately 77% of the participants were educators in school integration services or territorial educational services, while teachers accounted for 23% of the total. About 70% of the respondents worked with elementary school children, while the remaining 30% worked in secondary schools.

### Section D: multiple choice questions

In the multiple-choice questions in Section D, the first finding that stands out is that war metaphors (VS category) are never among the first two options. In general, for each question, the two most frequent answers belonged to categories other than VS. However, metaphors in the VS category rank well in almost all categories. They are the third option of questions on the following aspects of the pandemic: the vaccine, thought of as a “weapon of counterattack” (chosen by 35); the end of the pandemic as “regaining freedom” (by 45);^2^ the others as “allies” in the conflict against the virus, used by 50 of the participants. If we also interpret “refuge” as an option related to the war domain, we can say that the metaphor also has a main position in the question on measures to contain the contagion.

Among the aspects frequently described by war metaphors, we can also add Covid-19 (understood as the disease, the virus), since the metaphor of the enemy-conqueror is in the fifth position, but the numerical difference from previous positions is minimal. Something similar applies to the question about measures to contain the contagion, where 29 respondents chose “counterattack.” The questions in which the war frame option is relatively infrequent concern the pandemic as an overall situation (25), the spread of contagion (13), and doctors (23).

In both questions, where the war frame is frequently used and where war metaphors are chosen by a small number of respondents, the first two positions in the ranking are occupied by metaphors included in the TURN and/or FRONT experience categories, which evoke the positive and active attitudes of collaboration-sharing and projection toward the future. Among these, metaphors related to the frame of the game (FRONT) are very attractive: we find them in the first two positions to describe the pandemic as an overall situation (game), COVID-19 (opponent), and the spread of contagion (dominoes). The game option has a particularly strong appeal for describing measures to contain contagion, in which case it is the prevalent option (it is chosen by 83 respondents, 68% of the total number of participants, and the second option by numerosity stops at 45%).

Moreover, if the responses given by individual respondents are compared, it results that preferring the war frame for some features of the pandemic does not correlate with the choice of the same frame for the pandemic as an overall situation. For example, 21 chose to describe COVID-19 as the enemy, but not the pandemic as war. 36 respondents also considered the ally metaphor “suitable” for talking about others in the pandemic without describing the pandemic as an overall war situation. Also, in general, in the singular choice “war” goes along with the choice of different conceptual frames, both in the answer to the same question and in answers to other questions. The multiple-choice options were all accompanied by implications and attributes that clarified their conceptual meanings. Thus, it can be ruled out the possibility that this inconsistency may be owing to a misunderstanding or different interpretation of the proposed options. Instead, in our view, the variety of choices should be interpreted as indicating that metaphors are not necessarily a reflection of an overlying structural correspondence between the concepts involved. Conversely, if war is chosen for the option “Pandemic as an overall situation,” the option tends to be chosen for other aspects as well, but it does not prevent other options close to very different categories from being chosen for the same aspect.

In conclusion, the choice of the war metaphor is relatively frequent, but it is neither prevalent over the description frames, nor does it seem to constrain options and limit the variety and plurality of answers.

### Sections B and F: implicit metaphors

Other interesting results can be obtained looking at the responses to the open-ended questions in Sections B and F. Let us start with Section F. Those who selected the war option in the multiple choice, when asked for a further selection of metaphors to be used in the professional context,^3^ did not choose metaphors that could be placed in the VS category. In general, the metaphors proposed here are far from the frame of war (which is taken up by only a few respondents): the most mentioned metaphor here is again play/game; it is followed by the frame of change, revolution, and rebirth, a series of expressions that refer to the concept of union, sharing, and travel. Thus, the proposal of alternatives seems to stimulate further deviations from the frame of violent confrontation (see Table 2).

**Table 2:** “Among the metaphors proposed in this questionnaire, is there one or are there any that you find interesting and would like to use in educational activities?” (Section F).

Metaphorical words	Number of occurrences	Category
Game	17	FRONT
Change	9	TURN
Reborn	9	TURN
Trip	9	FRONT
Mountain	7	FRONT
Match	6	FRONT
Strategies	6	FRONT, VS
Revolution	5	TURN
Transformation	5	TURN
Win	5	FRONT
Restart	4	FRONT
Overcoming	4	FRONT
Alliance	3	VS
Boat	3	FRONT
Collaboration	3	FRONT
Mates	3	FRONT
Heroes	3	AROUND
Protagonists	3	FRONT
Renewal	3	TURN
Earthquake	3	AROUND

Counting of metaphorical words (more than three in number) in answers.

**Figure 1:**
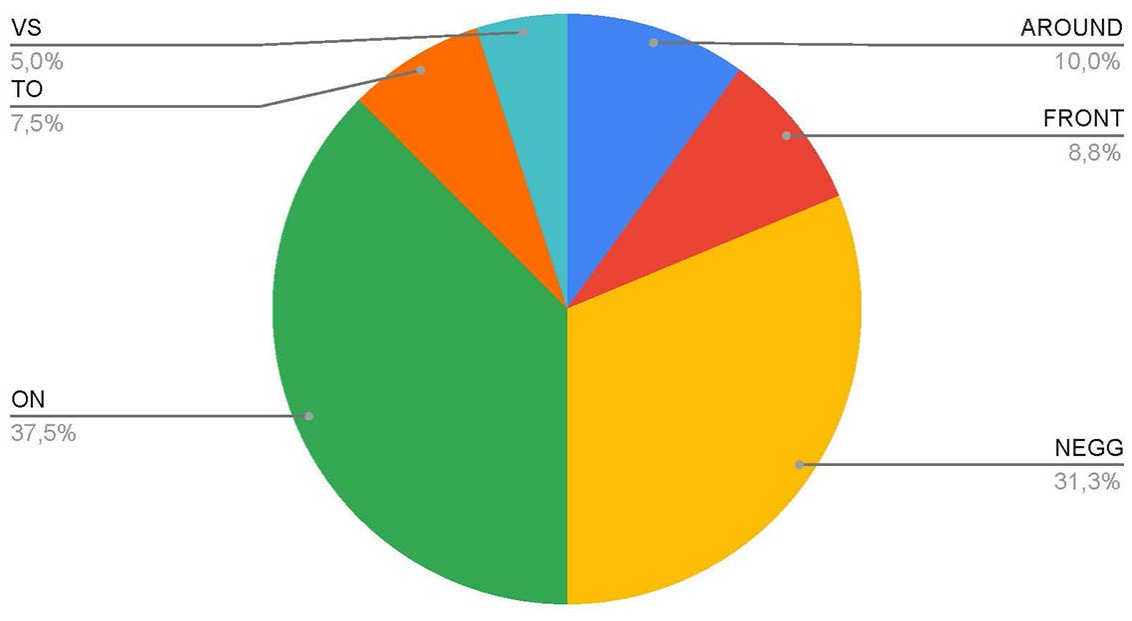
“What image comes to mind to describe the pandemic?” (Section B). Graph of percentages of answers for each category out of total metaphorical answers.

Additionally, the responses to the question in section B, asking generally for an image associated with a pandemic (without indication of professional use), show low appeal for the war frame among respondents that produce metaphors (Figure 1). Only four respondents chose metaphorical images adhering to the war frame. Images that can be linked to other frames prevail: 25 metaphorical images (about 20% of respondents; 37.5% of metaphorical images) can be linked to frames from the category named NEGG, which recalls the idea of negation as annihilation, radical subversion (“desert,” “bomb,” “madness”); 30 respondents propose metaphorical images that have a frame from the category named ON, which corresponds to the concept of limitation, constraint and partial deprivation. Even when respondents are questioned without referring to their work context and, thus, disregarding educational purposes, the frame of war and confrontation remains in the background. Furthermore, the prevailing frames of metaphoric images here are those of the ON category, which recall an idea of deprivation, limitation, and constraint rather than a constructive reaction and a horizon of change, as in the case of multiple choice; therefore, the request for free association seems to direct the imagination to the most negative aspects; they are nevertheless filtered through frames other than that of war.

Among the images in section B, the most “original” metaphorical images seem to be related to constructive and resilient reactions, among which many are true metaphors:

– Desert with small oases: “A place of loneliness where you find yourself alone and you have to find a way to survive and find small hidden oases where you can quench your thirst with energy.”
– The back of the turtle: “it gives me the idea of being strong, brave, and calm like a turtle.”
– Magnifying glass: “symbolizes research…scientific research, relational research, research of the other…symbolizes the change of perspective, seeing the issues around us in an amplified way.”
– People distant from each other but positioned in a circle: “while respecting the distances, I believe that collaboration and sharing between people is indispensable to overcome the bad period.”
– Puzzle:
– “like a puzzle, one thing, which is the class group, finds itself divided and more fragile.”
– A bubble:
– “you inside the bubble can see what is going on outside, but you find it hard to hear and be heard.”
– A chest of drawers, an object with various compartments: “pandemic is a container-word, which contains within it different aspects of contingent reality.”
– Mountain: “I see the pandemic as a big obstacle to overcome.”
– The darkest moment of the night [with clarification in the next answer:] for it is succeeded by the dawn, with its light revealing what the darkness hides.

### Effectiveness and limitations of methodological choices

– Our choice to associate conceptual options with their pragmatic implications has limitations and advantages. The association may influence choices, but it clarifies the meaning of the options to be chosen. Additionally, the implications we have specified can create rigidities that may not always be explanatory.
– The overall questionnaire was very extensive and articulated; it was supposed to facilitate the exercise of critical reflection, but it made it more onerous and laborious to complete, partly because of redundancies.
– Reliability of categories: unlike other studies (Gök and Kara, 2022; Sipahioglu, 2022), no further experts were contacted for the reliability test of metaphoric categories.
– The classification of options could be improved with regard to: ambiguities (e.g., the correspondence of one option to several categories); incoherence of some associated implications in brackets; genericity of the FRONT category (it should be divided into two categories, namely one to name the experience that finds expression in the metaphor of “play,” and thus implies collaboration, role assignment, sharing, rules; the other labeling the experience of dealing with a problem, an obstacle to be overcome); the absence of options for the ON category in the vaccine metaphors question.

## Discussion

Based on the analysis described above, below is a summary of the findings of the study:

– The overall metaphorical imagery that arises from the responses in section B differs from the imagery of conflict, despite being predominantly focused on the negative and pessimistic aspects of the pandemic.
– In the questions in section B asking for associations between words and images, the most original/creative metaphors provided correspond to positive and conscious attitudes rather than pure mirroring and representation. Positive attitudes were elicited more by images than by words.
– In questions about metaphors in educational language (section D), the choice of the war metaphor was neither prevalent nor exclusive, and choices oriented toward sharing and resilience were more frequent.
– There is a difference between spontaneous associations, expressions of experience in the first two answers, and metaphorical associations in the educational situation (multiple-choice options). In an educational context, the weight of the most negative/destructive options decreases.
– When choosing a single metaphor for an educational activity (in section F), this metaphor deviates completely from the war frame. Hypothesis: Once exposed to more alternatives through multiple choice options, the war frame is removed.

The general finding resulting from the analysis is that the war metaphor is not prevalent among the associations describing educators and teachers’ experiences, even where the experience is mainly negative. Based on the collected responses, it also appears that metaphors expressing resilient concepts and attitudes are mostly used in educational situations. Original metaphors are mostly correlated with the need to adopt a resilient attitude. Furthermore, the process of completing the questionnaire within the effort to understand and select a variety of metaphors seems to have pushed the most negative conflict frames further away.

The results provided support for the following hypotheses:

– The massive presence of the war metaphor in media communication and public discourse does not determine *per se* the preference for the war frame over other frames in expressing the experience of pandemic.
– The negative features of the pandemic experience are not necessarily correlated with the use of war frames. The frame does not appear to be the most frequent way to express negative feelings, and its choice does not depend directly on negative attitudes. Consequently, there seems to be no reason to argue that avoiding war metaphors should conversely favor a positive attitude.
– The choice of war metaphors for some pandemic features is not related to the exclusion of different metaphors. This result seems to be consistent with the findings of the aforementioned theoretical and empirical studies: The sensibility of the war frame does not imply that language use is homogeneous with the war frame. Thus, the chosen options cannot be explained as the declination of an unambiguous conceptual structure based on the network of implications of a single conceptual frame. This does not prove the lack of a coherent conceptualization of experience. Instead, it may support the idea that the pandemic, as an object of experience, is not a framed concept from which a network of implications branches, but a node of a plurality of projections and articulations.
– Growing awareness of the plurality and variety of metaphorical options for the features of the pandemic encouraged a critical and unassumed use of war metaphors, further obviating the potential persuasive power of the frame. In this regard, the study is in step with Wicke and Bolognesi (2020) when they write that… “a plethora of framing options—or a metaphor menu—may facilitate the communication of various aspects involved in the COVID-19-related discourse on the social media, and thus support civilians in the expression of their feelings, opinions and beliefs during the current pandemic.” It may apply to the war metaphor what applies to the use of “doubt” in arguments concerning the scientific aspects of the pandemic: doubt does not necessarily carry with it conspiracy ideology; removing doubt does not eliminate the risk of conspiratorial closure; instead, defending doubt can be useful precisely for building trust (Mohammed and Rossi, 2022).

## Conclusion

Predicated upon these specific findings, some possible and more general theoretical hypotheses can be advanced. The availability of a plurality of metaphorical options and the opportunity for a critical exercise on the conditions and implications of their situated use, contributed to the flexible use and conscious choice of metaphors. They seem to have been used as cognitive and imaginative resources, rather than as linguistic manifestations of rigid conceptual framing or as tools of their ideological construction. These results offer support for the theoretical hypothesis that a rigid framing effect, and thus the construction of a closed and uniform conceptual network such as pandemic = war, is a limiting rather than an ordinary condition. This can occur if there are several concomitant conditions: strong automatisms in the use of language, deliberate use for framing construction, absence of autonomous expressive and communicative needs of the recipients (owing to lack of knowledge of the object), and absence of caring or accountability relationships between the parties. Conversely, the need to express one’s experience effectively and the educational pragmatic purpose, together with the availability of a variety of options and exercises in the conscious use of language, support flexibility in the conceptual framing of experience and a tension toward the search for appropriate epistemic and pragmatic solutions.

Furthermore, the production of original metaphors by teachers and educators appears to correspond to the need to express, represent, and refer to something for which there is no predetermined definition. On this basis, it has been proposed the hypothesis that the original metaphor is used because it is necessary. This hypothesis is supported by Black’s seminal theory. Black argues that creative metaphors help to constitute the aspects of reality that “enable us to see” (Black, 1977, p. 454). Black compares creative metaphors to theoretical models, which allow scientists to understand an almost unknown object by attributing independent properties and unedited categories. Black argues that both creative metaphors and theoretical models operate in the identification between the object to be known and the medium, rather than a comparison based on analogical correspondences. The language adequate to the model is used for the new domain, so that inferences are not ruled by analogy but proceed “through and by means of an underlying analogy” (Black, 1962, pp. 228–229). Creative metaphors also generate unpredictable implications (Black, 1977, pp. 439–440) since interaction is not reducible to the comparison of terms in play; strong and active metaphors lead to an innovation of meanings that can be interpreted as the creation of new objects of knowledge and experience. Black argues that metaphorical thinking is the “embodiment” of a peculiar insight (Black, 1977, p. 448): through metaphorical identification it becomes possible to establish a “name” for and make sense of our experience.

## Data availability statement

The original contributions presented in the study are included in the article/Supplementary material, further inquiries can be directed to the corresponding author.

## Ethics statement

Ethical review and approval was not required for the study on human participants in accordance with the local legislation and institutional requirements. The patients/participants provided their written informed consent to participate in this study.

## Author contributions

The author confirms being the sole contributor of this work and has approved it for publication.

## Funding

The work was supported by the Fondo di Ateneo per la Ricerca (2021) of the Department of Education and Humanities of University of Modena and Reggio Emilia.

## Conflict of interest

The author declares that the research was conducted in the absence of any commercial or financial relationships that could be construed as a potential conflict of interest.

## Publisher’s note

All claims expressed in this article are solely those of the authors and do not necessarily represent those of their affiliated organizations, or those of the publisher, the editors and the reviewers. Any product that may be evaluated in this article, or claim that may be made by its manufacturer, is not guaranteed or endorsed by the publisher.

## Footnotes

^1^The meaning of the label names is clarified as follows: NEGG, with the double “G,” is the name for a strongly negative attitude; AROUND is the label for the idea of being surrounded by a wide-ranging phenomenon; ON is the label for the experience of a force, a power hanging and acting from above our level of control; *VS* is the label for the active conflict; the FRONT label refers to the experience of facing and trying to overcome an obstacle, an objective problem that is, precisely, in front of the subject; finally, the label named TURN is meant to indicate the experience of a turning point, of a change to a new condition.

^2^This metaphor is classified in the category of oppression-coercion, ON, but we took into account that it could also be interpreted by respondents in VS.

^3^The question: “Among the metaphors proposed in this questionnaire, is there one or are there any that seem interesting to you and that you would like to use in teaching activities?”
