# Supplementary material for: Exploring pandemic metaphors in educational contexts: a survey on the language of teachers and educators in Reggio Emilia, Italy
Source: Front Psychol. 2023 May 16;14:1192653. doi: 10.3389/fpsyg.2023.1192653 (PMC10227590; doi:10.3389/fpsyg.2023.1192653)
Supplement: Supplementary file 1 [file Table_1.docx]

Supplementary Material

Alice Giuliani

*** Correspondence:** Corresponding Author: [alice.giuliani@unimore.it](mailto:email@uni.edu)

Table 1: “What image comes to mind to describe the pandemic?” (section B, table for graph 1)

| **Category** | **Number of metaphorical images** | **Frequency (on metaphors) (%)** | **Frequency on respondents (%)** |
| --- | --- | --- | --- |
| ON | 30 | 37,5 | 24,6 |
| NEGG | 25 | 31,25 | 20,5 |
| AROUND | 8 | 10 | 6,6 |
| FRONT | 7 | 8,75 | 5,7 |
| TO | 6 | 7,5 | 4,9 |
| VS | 4 | 5 | 3,3 |
| Total of metaphorical images | 80 |  |  |
| Total of respondents | 122 |  |  |

Table 2, 3 4 5 6 7 8 9. Section D: Counting of multiple-choice answers and related categories.

| **Options for The pandemic as an overall situation:** | **Counting** | **Category** |
| --- | --- | --- |
| **Total** | **274** |  |
| Revolution, transformation (opportunity for change) | 64 +2 = 66 | TURN |
| Play (with rules, finding solutions, strategies...) | 49 +1(shangai)= 50 | FRONT |
| Match (to win, to compete in...) | 40 + 1(fight) = 41 | FRONT |
| Night, darkness, nightmare (which must end, to be brightened...) | 27 + 2 | NEGG |
| War (to fight, in which to eliminate the enemy...) | 25 | VS |
| Madness (chaos, imbalance) | 19 | NEGG |
| Storm (with ship at the mercy of the waves, boat in danger of sinking) | 16 + 2 | AROUND |
| Dictatorship (compromised freedoms, abuse of power, control) | 11 | ON |
| Fire (blazing, spreading, compromising, destroying) | 7 | AROUND |
| Acceleration toward what matters | 1 | TURN |
| Falling off the bike And getting back up | 1 | FRONT |
| Imbalance | 1 | NEGG |
| Invisible thread that connects us all even though we are far apart | 1 | AROUND (positive) |
| Teamwork | 1 | FRONT |
| I talked about the pandemic as a lived reality | 1 |  |
| Fight | 1 | FRONT |
| I haven't had a chance to talk about it | 1 |  |
| I didn't happen to use any | 1 |  |
| Night, as a dark moment that, however, has an end at dawn | 1 | NEGG |
| New perspectives and viewpoints, change of focus, change of observation point, change of patterns, nature re-emerging | 1 | TURN |
| Storm clouds rolling in on us (from which we can protect ourselves but of which we have no control) | 1 | AROUND |
| Challenge | 1 | FRONT |
| Challenge: to our ability to do things within the rules, for the good of ourselves and others | 1 | FRONT |
| Shangai: a complexity of elements that must be untangled to be understood and made one's own in one's cultural background. | 1 | FRONT GAME |
| Situation "to be controlled" | 1 |  |

| **Options for The Covid-19** | **Counting** | **Category** |
| --- | --- | --- |
|  | **Total: 243** |  |
| Alarm/messenger (waking us up, reporting global problems) | 48+1 | TURN |
| Opponent (in a game, which has its own goal, on which we must prevail) | 45 | FRONT |
| Mountain (obstacle to overcome) | 38+1 | FRONT |
| Monster, ghost (unseen, looming, scary) | 37 | NEGG |
| Enemy, conqueror (to defend against, to react against) | 35 | VS |
| Blow/hammer/shock (shaking, knocking down, destabilizing) | 23 | NEGG |
| Flame (seeking fuel, burning) / rain (accumulating, finding cracks, seeping) | 5 | AROUND |
| Explosive device (to defuse, to render harmless) | 5 | VS |
| As a thing (epidemic) that in human history has cyclically recurred | 1 |  |
| I mentioned Covid-19 as a contagious disease. | 1 |  |
| Temporary illness, like many others that have been defeated over time and for which a cure has been found | 1 |  |
| I haven't had a chance to talk about it | 1 |  |
| I didn't happen to use any | 1 |  |
| PROBLEM TO BE SOLVED | 1 | FRONT |
| Stop that made us stop and think, forced us to stop and observe the frenzied destruction we are bringing to nature through pollution and waste. | 1 | TURN |

| Options for The spread of contagion as | Counting | Category |
| --- | --- | --- |
| Total | 199 |  |
| Domino, chain reaction, word of mouth (to break, interrupt) | 45 | FRONT |
| River breaking banks (event to be prevented, depending on errors) | 32 | FRONT |
| Earthquake (which shakes the ground underfoot, takes away stability, creates insecurity) | 32 | NEGG |
| Avalanche, tsunami-flood (overwhelming, unpredictable, uncontainable...) | 26 | AROUND+1 |
| Fire (to be contained, whose damage to be reduced) | 19 | AROUND |
| Colonization (of parasites, aliens...) | 14 | ON |
| Military attack, military campaign (large, spread over several fronts, with organized troops) | 13+1 | VS |
| Train derailing (event to be prevented, depending on errors) | 9 | TURN |
| We spoke to them without metaphors, explaining it scientifically | 1 |  |
| The virus likes to travel inside our droplets. Together with us it comes to school, rests on desks, doesn't like gel.... If we scream without a mask it makes long long jumps | 1 |  |
| Cross time bomb | 1 | VS |
| Consequence | 1 |  |
| Liquid spilling and expanding | 1 | AROUND |
| I haven't had a chance to talk about it | 1 |  |
| I didn't happen to use any | 1 |  |
| I don't remember | 1 |  |
| Obstacle to normality | 1 |  |

| **Options for Measures to contain contagion** | **Counting** | **Category** |
| --- | --- | --- |
| **Total** | **245** |  |
| Role play/group work (collaboration) | 83 | FRONT |
| Pause-suspension, parenthesis (from the ordinary, to reflect, return to self) | 37 | FRONT |
| Shelter/shelter (in which to stay safe) | 33 | AROUND/VS |
| Collective experiment (we are not sure of the results, we go by trial and error) | 31 | FRONT |
| Counterattack, resistance (to oppose, not to be annihilated, defeated) | 29 | VS |
| Prison (helplessness, absence of freedom, physical constraint) | 13 | ON |
| Abyss, tunnel (no light, no exit in sight) | 7 | NEGG |
| Seclusion, asceticism (revealing hidden, previously invisible things) | 7 | TURN/NEGG |
| "the virus must not travel" (I would like to point out that the teachers in the class in which I work did not want to address contagion, pandemic, etc. At all; these are my observations during informal times with the children) | 1 |  |
| Collective experiment (with positive meaning, belief in science, proceeding with experiments based on observation and study) | 1 | FRONT |
| I didn't happen to use any | 1 |  |
| Test of responsibility and solidarity | 1 | FRONT |
| A way to protect ourselves and others | 1 |  |

| **Options for The others during the pandemic:** | **Counting** | **Category** |
| --- | --- | --- |
|  | 277 |  |
| Companions (of adventure, travel, in the same boat) | 79 | TURN / FRONT |
| Support, source of energy (to move forward, to face difficulties, to start again) | 65 | FRONT |
| Allies (in the conflict against the virus) | 50 | VS |
| Lead actors (all have mission, important role) | 44 | FRONT |
| Masks/aliens (we do not see their faces) | 10 | NEGG |
| Puppets, marionettes (at the mercy of others' decisions) | 8 | ON |
| Threat / anointers / spies (someone to be wary of) | 8 | VS |
| Missing (whom we have lost track of, whom we cannot meet) | 7 | AROUND |
| Pawns (to be placed, organized in a strategy) | 2 | FRONT |
| "It's good to be together with the other group." | 1 |  |
| I didn't happen to use any | 1 |  |
| I didn't talk about it in any of these terms | 1 |  |
| People like us | 1 |  |

| **Options for The physicians and health personnel active in the care of Covid-19:** | **Counting** | **Category** |
| --- | --- | --- |
|  | 229 |  |
| Guides (who explore, lead us to the way out...) | 55 | FRONT |
| Angels (who guard, protect...) | 40 | NEGG |
| Heroes, superheroes (with above-average talents, capable of measuring themselves against abnormal events) | 29 | AROUND |
| New protagonists (who were in the shadows, coming to the foreground compared to other characters) | 29 | FRONT |
| Soldiers (in the war on the virus) | 23 | VS |
| Machines (tireless, working non-stop) | 21 | FRONT |
| Victims (sacrifice themselves for the good of the collective) | 10 | NEGG |
| Agents/special agents (in a dictatorship, of the new power of medicine and science) | 6 | ON |
| Judges (decide life/death) | 4 | ON |
| Stars (who love notoriety, who seek prominence) | 2 | ON |
| Officers doing their duty | 1 |  |
| Experts, who can play a specific part in this collective effort because of their studies and competenzr | 1 |  |
| I haven't had a chance to talk about it | 1 |  |
| I didn't happen to use any | 1 |  |
| There was no mention of health personnel | 1 |  |
| People who do their work | 1 |  |
| People who make their time, skills and expertise available to others without wanting anything in return | 1 |  |
| People particularly inclined to help and care for each other | 1 |  |
| Support, reference, model | 1 |  |
| They carry out the task they have chosen | 1 |  |

| **Options for The End of the Pandemic:** | **Counting** | **Category** |
| --- | --- | --- |
|  | **278** |  |
| Restart (after overcoming an obstacle) | 82 | FRONT |
| Rebirth, renewal | 66 + 2= 68 | TURN |
| Regaining freedom | 45 | VS / ON |
| Liberation (from an invasion, occupation) | 36 | VS |
| Return of light, miracle | 21 | NEGG |
| Salvation / victory (find escape) | 13 | AROUND/ON7VS |
| Oasis/mirage (which could be an illusion) | 10 | NEGG |
| "When does it all end, our group is together with the other group?" | 1 |  |
| I didn't happen to use any | 1 |  |
| I don't know | 1 |  |
| Breathing freely, appreciating everything even more | 1 | TURN |
| Some newfound lightness | 1 | TURN |

| **Options for The vaccine as product of scientific research** | **Counting** | **Category** |
| --- | --- | --- |
|  | **221** |  |
| Turning point (in the path of change initiated by the pandemic) | 53 | TURN |
| Neutralizer, tamer (which is supposed to make the virus less dangerous, allows you to live with it) | 38 | AROUND |
| Counterattack weapon (against the virus attack, to overcome its "troops") | 35 | VS |
| Way out, "esc" key (from the scope of the virus) | 32 | NEGG |
| Strategy, trick (to boycott the virus, weaken it) | 21 | FRONT |
| Trainer (to instruct our body to react) | 18 | FRONT |
| Gatekeeper - filter (which prevents the virus from hitting us, makes it wait) | 15 | FRONT |
| Help | 1 |  |
| Achievement and social symbol | 1 |  |
| I haven't had a chance to talk about it | 1 |  |
| I didn't happen to use any | 1 |  |
| WE HAVEN'T TALKED ABOUT IT | 1 |  |
| I am not sure if it is effective. | 1 |  |
| Important discovery | 1 |  |
| Just one proposal, among other possible ones. | 1 |  |
| Vaccine intended as a spell/desire that makes the coronavirus disappear | 1 |  |
